# States Transitions Inference of Postpartum Depression Based on Multi-State Markov Model

**DOI:** 10.3390/ijerph18147449

**Published:** 2021-07-13

**Authors:** Juan Xiong, Qiyu Fang, Jialing Chen, Yingxin Li, Huiyi Li, Wenjie Li, Xujuan Zheng

**Affiliations:** Health Science Center, Shenzhen University, Shenzhen 518060, China; jxiong@szu.edu.cn (J.X.); fangqiyu320@szu.edu.cn (Q.F.); chenjialing0829@163.com (J.C.); Wuwu009lyx@163.com (Y.L.); 18576627549@163.com (H.L.); 2019227013@szu.edu.cn (W.L.)

**Keywords:** multi-state Markov model, postpartum depression, transition probability, proactive prevention

## Abstract

*Background*: Postpartum depression (PPD) has been recognized as a severe public health problem worldwide due to its high incidence and the detrimental consequences not only for the mother but for the infant and the family. However, the pattern of natural transition trajectories of PPD has rarely been explored. *Methods:* In this research, a quantitative longitudinal study was conducted to explore the PPD progression process, providing information on the transition probability, hazard ratio, and the mean sojourn time in the three postnatal mental states, namely normal state, mild PPD, and severe PPD. The multi-state Markov model was built based on 912 depression status assessments in 304 Chinese primiparous women over multiple time points of six weeks postpartum, three months postpartum, and six months postpartum. *Results*: Among the 608 PPD status transitions from one visit to the next visit, 6.2% (38/608) showed deterioration of mental status from the level at the previous visit; while 40.0% (243/608) showed improvement at the next visit. A subject in normal state who does transition then has a probability of 49.8% of worsening to mild PPD, and 50.2% to severe PPD. A subject with mild PPD who does transition has a 20.0% chance of worsening to severe PPD. A subject with severe PPD is more likely to improve to mild PPD than developing to the normal state. On average, the sojourn time in the normal state, mild PPD, and severe PPD was 64.12, 6.29, and 9.37 weeks, respectively. Women in normal state had 6.0%, 8.5%, 8.7%, and 8.8% chances of progress to severe PPD within three months, nine months, one year, and three years, respectively. Increased all kinds of supports were associated with decreased risk of deterioration from normal state to severe PPD (hazard ratio, HR: 0.42–0.65); and increased informational supports, evaluation of support, and maternal age were associated with alleviation from severe PPD to normal state (HR: 1.46–2.27). *Conclusions:* The PPD state transition probabilities caused more attention and awareness about the regular PPD screening for postnatal women and the timely intervention for women with mild or severe PPD. The preventive actions on PPD should be conducted at the early stages, and three yearly; at least one yearly screening is strongly recommended. Emotional support, material support, informational support, and evaluation of support had significant positive associations with the prevention of PPD progression transitions. The derived transition probabilities and sojourn time can serve as an importance reference for health professionals to make proactive plans and target interventions for PPD.

## 1. Introduction

Postpartum depression (PPD) is defined as a non-psychotic depressive episode beginning in or extending to the postpartum period [1]. In addition to the similar symptoms of depression at other times of life such as loss of interest, insomnia, irritability, exhaustion, poor concentration, low self-respect, and thoughts of suicide, women who have PPD also experience guilty feelings about taking care of their new baby [2,3,4,5]. Due to the high incidence worldwide, PPD has been recognized as a severe public health problem during the last decades [3,6]. Longitudinal and epidemiological studies have found the at the prevalence of PPD in different countries varied from 10% to more than 25% in the first year postpartum [7,8,9]. A systematic review reported that the average prevalence of PPD in developed countries was 19.2% among mothers [10], while in low- and middle-income countries, up to 20–25% of women were found to suffer from PPD [11].

In addition to its high incidence, PPD can lead to several detrimental consequences in the longer terms not only for the mother, but for the infant and the family as a whole [12]. First, PPD is associated with the hazards for the mother’s mental and physical wellbeing [3,13]. PPD was found to be one of the leading causes of maternal morbidity and mortality in various countries [3]. Second, PPD is related to the impairment of mother–infant attachment [14] and maternal self-efficacy [15,16], which could lead to the longer-term disruption of motional and cognitive development of the infant [14,17]. Additionally, women with PPD are more likely to stop breastfeeding earlier than non-depressed mothers [18,19]. Third, PPD has an adverse effect on the marital relationship and the psychological health of the spouse [20]. For instance, PPD had a considerable burden on close family members, affecting social and leisure activities and posing financial challenges within the family [17].

Extensive studies have identified various risk factors of PPD including biological and psychological categories [21,22]. Biological risk factors of PPD included the endocrine system, the immune system, and genetic factors [23,24,25]. Psychosocial factors involved previous history of depression and anxiety, marital status, stressful status, social support, etc. [26,27]. From the perspective of risk factor discovery, regression and structural equation analysis have been widely used in PPD studies to explore the factors [3,15,16,28]. However, the above analysis offers little information about the dynamic process of disease evaluation [29]. 

In the existing literature, the pattern of nature progressive trajectories of PPD from normal status has been poorly explored. Therefore, it would be worth investigating its natural progression for early detection and prevention of PPD. It is noted that multi-state Markov modeling is a modeling technique that has been widely used in the medical field to analyze the progression of a variety of diseases such as high blood pressure, diabetes, and chronic kidney disease [29,30,31]. Likewise, it can be used to analyze the transition from a normal (non-disease) state, through a mild state to a severe and/or death state [32]. In this study, we were interested in estimating the average transition rates between depression states, and also wanted to investigate potential factors related to the rates of transition. The depression statuses are represented as Markov states, and the depression status changes are shown as transition probabilities between states. Thereby, a multi-state Markov model was used in this research to explore the PPD progression process, providing information on the transition probability, hazard ratio, and the mean sojourn time in each state to fill the research gap. 

## 2. Materials and Methods

### 2.1. Study Design and Participants

**Figure 1:**
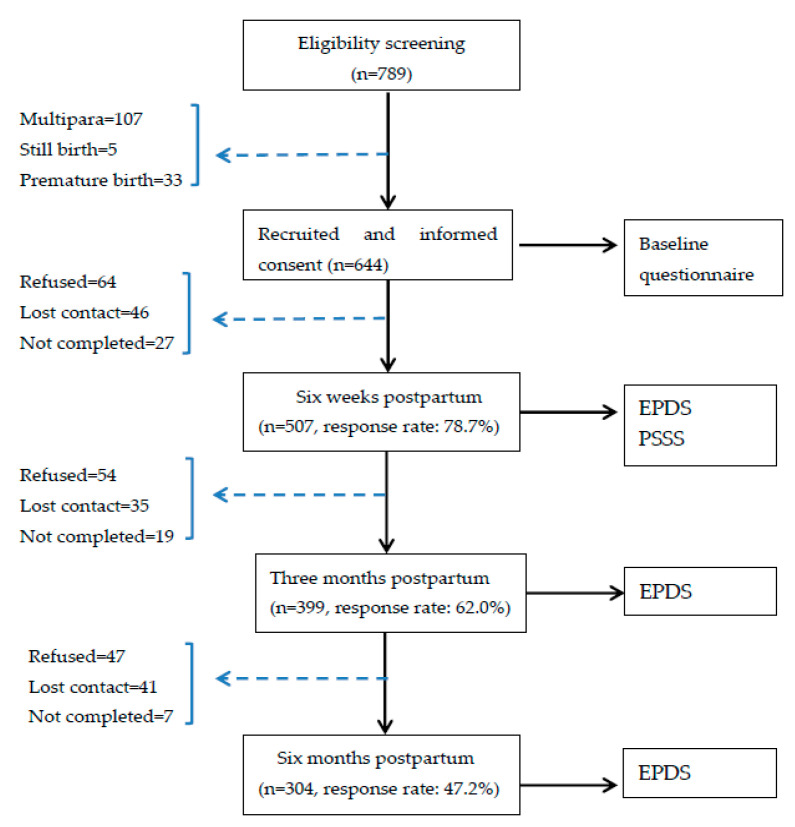
Flow of participants through the study. EPDS: Edinburgh Postnatal Depression Scale; PSSS: Postpartum Social Support Scale.

A quantitative longitudinal study was conducted to uncover the state transitions of PPD based on the multi-state Markov model among Chinese primiparous women. Ethical approval of this research was conducted in the Health Science Center at Shenzhen University. Women were eligible to enroll in the study if they were married; aged 18 years or above; first-time mothers with healthy baby; and able to respond to the questionnaires. Women were excluded from the study if they or their infants had serious diseases. From January to December 2020, a total of 644 participants were recruited in the Department of Obstetrics and Gynecology of the three public hospitals in China. Flow of participants through the study is illustrated in Figure 1. Before data collection, all participants provided written informed consent and were informed of the purpose of the study and freedom to withdraw at any time in the research process.

### 2.2. Measurements

Data on maternal age, marital status, maternal education, occupation, family income (per person, month), mode of childbirth, whether attending parenting training, baby gender, baby health, and baby fussiness via self-report by new mothers were collected by baseline questionnaires developed by the researchers. 

The researchers measured the depressive symptoms by the Edinburgh Postnatal Depression Scale (EPDS) [33], which is the most widely used screening tool for PPD, and translated into over 60 different languages [34]. EPDS is a comprehensive, easy to administer, self-reporting scale of ten short items, where responses are scored from 0 to 30 based on the symptom severity. The higher score indicates the worse mental health status women have. The reported Cronbach’s alpha coefficient of Chinese version EPDS was 0.87, and its concurrent validity with the BDI (Beck Depression Inventory) was 0.79 [35]. In the present study, the internal consistency of the EPDS was 0.87. According to the maximized combined sensitivity and specificity of this tool, a 0–9 score was categorized as “normal state”, scores of 10–12 were categorized as “mild PPD”, and those more than 13 were listed as “severe PPD” in mainland China [35,36,37]. 

Postpartum Social Support Scale (PSSS) in the Chinese version was used to assess the various supports received by primiparous women after childbirth. The 20-item instrument includes four kinds of postnatal support including emotional support (five items), material support (five items), informational support (five items), and evaluation of support (five items). The scale uses the 4-point Likert score type and ranges from 0 to 3 points depending on the response options of “never”, “rarely”, “sometimes”, and “often”. A higher score the mother obtains, the more postnatal support she receives. The Cronbach’s alpha coefficient of this tool was 0.89 [38]. The internal consistency of PSSS was 0.90 in the current research.

### 2.3. Data Collection Procedure

Baseline questionnaires and contact details of participants were collected in the Department of Obstetrics and Gynecology by the research team face to face on the third to the fifth day postpartum. The electronic documents of EPDS and PSSS were sent to participants via WeChat or email at six weeks postpartum. The follow up electronic EPDS was likewise distributed to these women at three months postpartum, and six months postpartum, respectively. The WeChat or call reminders were given to participants before and after one week, and one day of the three different time points to improve the response rate.

### 2.4. Data Analysis 

Descriptive statistics were used to describe the social-demographic characteristics and clinical variables by mean (M), standard deviation (SD), and frequency proportions. Hazard ratios (HRs) and corresponding 95% confidence intervals (CIs) were used in the statistical analysis. The multi-state Markov model was built using the msm package of R software.

**Figure 2:**
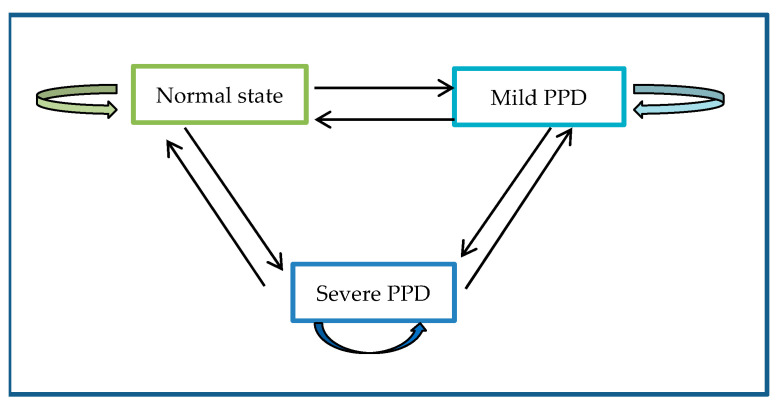
Three PPD state transitions in the Markov model.

In the Markov model, there were three PPD states of interest: state 1 (normal state), state 2 (mild PPD), and state 3 (severe PPD). Figure 2 illustrates nine possible transitions among the three transient states. For example, a new mother can stay in the current state or transit to any of the other states such as normal → normal, normal→ mild, normal → severe, mild → mild, mild → severe, mild → normal, severe → severe, severe → mild, severe → normal. The sojourn time means the average length of time staying in a transient state before transiting to a new state.

## 3. Results

### 3.1. Social-Demographic and Clinical Characteristics of the Participants

There were 304 participants who completed the questionnaires at six weeks postpartum, three months postpartum, and six months postpartum. These social-demographic and clinical characteristics of primiparous women are summarized in Table 1. For example, the average age of these participants was 24.8 (2.89) years and all of them were married.

### 3.2. Observed Numbers of PPD Status Transitions from One Visit to the Next Visit

Among the 912 depression status assessments in the 304 women over the period 6–24 weeks postpartum, 304 measurements were taken in the initial visit (T1: six weeks postpartum) and 608 measurements were taken at follow-up visits (T2: three months postpartum, T3: six months postpartum). Table 2 shows the number of transitions from one visit (given by the row state) to the next one (given by the column state). Of the 608 transitions, 6.2% (38/608) showed deterioration of mental status from the level at the previous visit, while 40.0% (243/608) showed improvement at the next visit, and 53.8% (327/608) showed no change. For example, there were 155 transitions where the participant had no PPD (normal state) and remained in the normal state at the next visit, and nine cases where a participant in normal state developed mild PPD at the next visit. There were ten cases where participants in the normal state developed severe PPD. Similarly, there were 33 occurrences where participants remained in mild PPD and 19 cases where participants progressed from mild PPD to severe PPD at the following visit.

### 3.3. PPD State Transition Probabilities

Table 3 presents the model estimated probability of the next transition if and when a subject transitions from the current state at an examination, and the estimated sojourn time while in each state. For example, a subject in normal state who does transition then has a probability of 49.8% of worsening to mild PPD, and 50.2% to severe PPD. The subject with mild PPD who does transition has an 80.0% chance of improving to normal state or 20.0% chance of worsening to severe PPD. A subject with severe PPD is more likely to improve to mild PPD than developing to the normal state. On average, subjects remained in a normal state for 64.12 weeks before transitions to other states (sojourn time), remained in mild PPD for 6.29 weeks, and in severe PPD for 9.37 weeks.

### 3.4. Model-Estimated Transition Probabilities over a Given Follow-Up Interval

**Figure 3:**
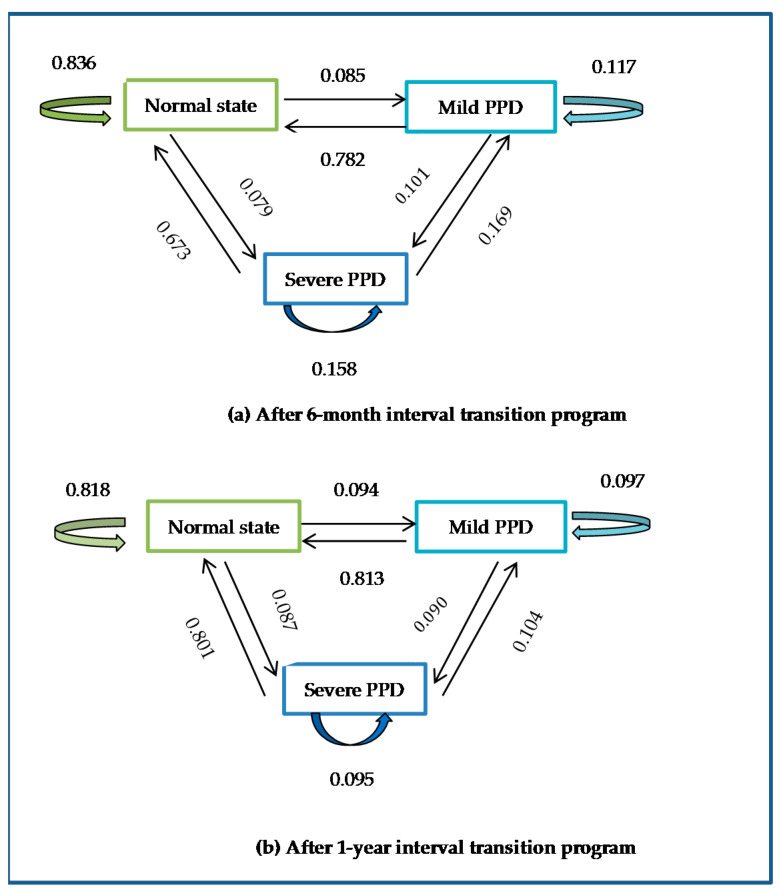
State transition diagrams.

On the basis of the probabilities of transitions from state to state, the researchers computed the probabilities over a given follow-up interval of deterioration progress (state 1 through 2 to state 3) and alleviation progress (state 3 through 2 to state 1). Probability over a follow-up interval of one month to three years is shown in Table 4 and Figure 3. For example, women in normal state (state 1) had 6.0%, 8.5%, 8.7%, and 8.8% chance of progress to severe PPD (state 3) if the next checkup was performed after the interval of three months, nine months, one year, and three years, respectively. In contrast, women with severe PPD had 40.7%, 76.9%, 80.1%, and 81.6% chance of alleviation to normal state within three months, nine months, one year, and three years, respectively. 

### 3.5. Covariate Effects and Patient-Specific Risks

The covariates in the research included maternal age, marital status, maternal education, occupation, family income (per person, month), mode of childbirth, whether attending parenting training, baby gender, baby health, baby fussiness, emotional support, material support, informational support, and evaluation of support. The effects of covariates on PPD transition intensity with statistical significance are summarized in Table 5 and Table 6. Emotional support, material support, informational support, evaluation of support, and maternal age had some significant unadjusted associations with possible transitions. For example, increase in all kinds of support was associated with decreased risk of deterioration from the normal state (state 1) to severe PPD (state 3), and increased informational supports and evaluation of support were associated with alleviation from severe PPD to normal state. 

## 4. Discussion

Due to the high incidence and the severe consequence for mothers, infants, and families, PPD has become a severe public health problem worldwide during the last decades [3,6,12]. Thus, it was of significant importance to explore transition patterns in different PPD states and the risk factors relating to postnatal mental status alleviation and deterioration. Previous studies have investigated that primiparous women were more prone to suffer from PPD than multipara, and therefore worthy of more attention and investigation [15,16]. In the current research, a time-homogeneous continuous Markov model was used to explore the three PPD state transition probabilities, the mean sojourn time in each state, and the risk factors affecting the progression or regression of PPD in Chinese primiparous women, which has not been explored in the existing literature.

The present study found that of the 608 transitions, 6.2% (38/608) showed deterioration of PPD state from the level at the previous visit. In contrast, 40.0% (243/608) showed alleviation of PPD state at the next visit. It was consistent with the previous studies that indicated PPD symptoms in some women would remit with the passage to time [9,10,39]. However, a subject in normal state who does transition was found to have a probability of 49.8% of worsening to mild PPD and 50.2% to severe PPD; a subject with mild PPD had 20.0% chance of worsening to severe PPD; and a subject with severe PPD was more likely to improve to mild PPD than developing to a normal state. The results of PPD state transition probabilities have caused more attention and awareness about regular PPD screening for postnatal women and the timely intervention for women with mild or severe PPD. Health agencies in developed countries such as in the USA and the EU have recommended the regular screening of postpartum women to detect PPD symptoms [40,41,42].

Additionally, the transition probabilities over a 1-month to 3-year period were first estimated in the study. For instance, women in the normal state had 6.0%, 8.5%, 8.7%, and 8.8% chance of progress to severe PPD within three months, nine months, one year, and three years, respectively. Even though women with normal state had a low probability of developing PPD in the first postpartum year, the probability of maternal depression will increase substantially within three years. The findings well align with a prior study that found that PPD might persist for up to three years after childbirth [43]. The previous research showed approximately 25% of women had depressive symptoms at some point in the three years postpartum, and some women had increasing depressive symptoms over the three years [43]. Therefore, the preventive actions on PPD should be conducted at the early stages and three yearly; at least one yearly checkup or screening is strongly recommended, not merely during the period of pregnancy and postpartum visiting [44]. 

In terms of covariate effects, increase in all kinds of social supports was associated with decreased risk of deterioration from normal state to severe PPD (HR: 0.42–0.65) and increased informational supports and evaluation of support were associated with alleviation from severe PPD to the normal state (HR: 1.59–2.27). The previous research likewise identified that social support significantly affects the postnatal mental status for new mothers [15,16,45]. However, Chinese first-time mothers were reported to receive insufficient social support after childbirth, and particularly lacked adequate informational and appraisal support from health professionals [15,16]. It was almost not available for single support provider to offer all kinds of support [9]. Women’s family and friends are expected to offer them great emotional support and material support such as love, trust, time, and money; and health professionals could be appropriate to supply women with much more informational support and evaluation of support (i.e., professional advice and instructions on PPD prevention and treatment) [9,45]. Thus, in order to improve mental health outcomes, the family members of women and health professionals should supply new mothers with increased support to improve outcomes. Health professionals also need to play a more active and significant role in PPD alleviation transitions by offering professional informational and appraisal support. 

In summary, there are key contributions in the research. First, a multi-state Markov model was used to explore the state transition dynamics of three PPD states and investigate how various support types affected the progression or alleviation of PPD in the Chinese population. Furthermore, through the developed model, the sojourn time in each PPD state and the transition probabilities from one state to other state were estimated, thereby providing statistical foundations for regularly screening and time targeted interventions. However, some limitations of this study can be summarized as follows. First, recognizing the belief of “domestic shame should not be made public” [46], the percent of women with PPD could be underestimated. Second, women’s lifestyles may change dramatically over such a long period, which could violate the model’s assumption on time homogeneity, and it could lead to biases. Third, the potential effect of confounding covariates or interaction among covariates was not investigated in this study. Fourth, EPDS was the only screening tool of PPD used in the research, and the other commonly used depression measures such as the Beck Depression Inventory (BDI) and Montgomery-Asberg Depression Rating Scale (MADRS) could be used to test the results in further study. 

## 5. Conclusions

The results of the PPD state transition probabilities have created more attention and awareness about regular PPD screening for postnatal women and the timely intervention for women with mild or severe PPD. The preventive actions on PPD should be conducted at the early stages and three yearly; at least one yearly checkup or screening is strongly recommended, not merely during the period of pregnancy and postpartum visiting. All kinds of support including emotional support, material support, informational support, and evaluation of support had significant positive associations with the prevention of PPD progression transitions. The derived transition probabilities and sojourn time can serve as an importance reference for health professionals to make proactive plans and targeted interventions for PPD.

## Figures and Tables

**Table 1:** Social-demographic and clinical characteristics of the participants.

Variables	Total (*n* = 304)
Age, mean (SD)	24.8 (2.89)
Marital status, *n* (%)	
Married	304 (100.0%)
Divorced	0 (0.0%)
Single	0 (0.0%)
Education, *n* (%)	
Middle school or lower	78 (25.6%)
High school or junior college	123 (40.5%)
University or higher	103 (33.9%)
Occupation, *n* (%)	
Professional	10 (3.3%)
Skilled	25 (8.2%)
Unskilled	181 (59.5%)
Unemployed	88 (29.0%)
Family income (per person, month), *n* (%)	
<3000 yuan (US$420)	73 (24.0%)
3001—5000 yuan (US$420–700)	144 (47.4%)
>5000 yuan (US$700)	87 (28.6%)
Delivery mode, *n* (%)	
Natural childbirth	218 (71.7%)
Assisted childbirth	46 (15.1%)
C-section	40 (13.2%)
Whether attending parenting train, *n* (%)	
Yes	157 (51.6%)
No	147(48.4%)
Baby gender, *n* (%)	
Boy	181 (59.5%)
Girl	123 (40.5%)
Baby health, mean (SD)	80.5 (15.49)
Baby fussiness, mean (SD)	69.6 (19.89)
Emotional support, mean (SD)	10.2 (2.72)
Material support, mean (SD)	9.9 (3.43)
Informational support, mean (SD)	6.8 (3.14)
Evaluation of support, mean (SD)	8.4 (2.92)

**Table 2:** Observed number of PPD transitions from one visit to the next visit.

From\To	Normal State *n* (%)	Mild PPD *n* (%)	Severe PPD *n* (%)
T1 Normal state	155(25.5%)	9 (1.5%)	10 (1.6%)
T2 Mild PPD	88 (14.5%)	33 (5.4%)	19 (3.1%)
T3 Severe PPD	63 (10.3%)	92 (15.1%)	139 (22.9)

Note: State 1: Normal state (EPDS < 10); State 2: Mild PPD (EPDS ≥ 10); State 3: Severe PPD (EPDS ≥ 13); Time points: T1 (6 weeks), T2 (3 months), T3 (6 months). PPD: Postpartum depression; EPDS: Edinburgh Postnatal Depression Scale.

**Table 3:** PPD state transition probabilities.

From\To	Normal PPD	Mild PPD	Severe PPD
Normal state	-	0.498	0.502
Mild PPD	0.800	-	0.200
Severe PPD	0.064	0.936	-
Sojourn Time (weeks)	64.12	6.29	9.37

Note: The sojourn time means the average length of time staying in a transient state before transiting to a new state.

**Table 4:** Model-estimated transition probabilities over a given follow-up interval.

Interval of Follow-Up	State 1 to State 3 Percent (95% CI)	State 2 to State 3 Percent (95% CI)	State 3 to State 1 Percent (95% CI)	State 2 to State 1 Percent (95% CI)
1 month	0.028 (0.015, 0.059)	0.085 (0.050, 0.149)	0.104 (0.086, 0.143)	0.390 (0.332, 0.445)
3 month	0.060 (0.036, 0.108)	0.114 (0.080, 0.171)	0.407(0.351, 0.473)	0.680 (0.600, 0.735)
6 month	0.079 (0.050, 0.144)	0.101 (0.073, 0.157)	0.673(0.601, 0.721)	0.782 (0.700, 0.826)
9 month	0.085 (0.054, 0.150)	0.093 (0.063, 0.152)	0.769 (0.681, 0.818)	0.806 (0.709, 0.859)
1 year	0.087 (0.057, 0.161)	0.090 (0.059, 0.162)	0.801 (0.694, 0.851)	0.813 (0.700, 0.866)
2 year	0.088 (0.054, 0.149)	0.088 (0.054, 0.149)	0.816(0.712, 0.873)	0.816 (0.712, 0.873)
3 year	0.088 (0.056, 0.147)	0.088 (0.056, 0.147)	0.816 (0.718, 0.871)	0.816 (0.718, 0.871)

Note: Interval of follow-up refers to the time to the next examination for women in a given state at the beginning of the interval. State 1: Normal state (EPDS < 10); State 2: Mild PPD (EPDS ≥ 10); State 3: Severe PPD (EPDS ≥ 13).

**Table 5:** The effects of covariates on PPD worsening transition.

Worsening Transition	Hazard Ratio (95% CI)
State 1 to State 3	
Emotional support	0.48 (0.33, 0.70)
Material support	0.65 (0.51, 0.82)
Informational support	0.57 (0.42, 0.76)
Evaluation of support	0.42 (0.22, 0.82)

Note: State 1: Normal state (EPDS < 10); State 2: Mild PPD (EPDS ≥ 10); State 3: Severe PPD (EPDS ≥ 13).

**Table 6:** The effects of covariates on PPD bettering transition.

Bettering Transition	Hazard Ratio (95% CI)
State 3 to State 1	
Informational support	1.59 (1.15, 2.19)
Evaluation of support	2.27 (1.12, 4.58)
Maternal age	1.46 (1.15, 1.86)
State 3 to State 2	
Evaluation of support	1.14 (1.04, 1.26)

Note: State 1: Normal state (EPDS < 10); State 2: Mild PPD (EPDS ≥ 10); State 3: Severe PPD (EPDS ≥ 13).

## Data Availability

The data presented in this study are available on request from the corresponding author. The data are not publicly available due to privacy restrictions.
